# Radical surgical resection of giant Angiosarcoma of the posterior Mediastinum: A rare neoplasm with rare presentation as epigastric pain

**DOI:** 10.1016/j.amsu.2021.103087

**Published:** 2021-11-19

**Authors:** Ikram ul haq Chaudhry

**Affiliations:** Division of Thoracic Surgery Dammam Medical Complex, Dammam, 31444, Saudi Arabia

**Keywords:** Angiosarcoma, Mediastinum, Surgery, Epigastric pain

## Abstract

Angiosarcoma of the Posterior Mediastinum is a rare entity. We herein report a case of a giant posterior mediastinal Angiosarcoma. A 54-year female presented with a one-year history of epigastric pain. Her upper gastrointestinal tract endoscopy revealed no abnormality. Chest x-Ray showed a shadow in the hilar area. A computed tomographic scan of the Thorax (CT) and MRI Scan showed a mass in the Posterior Mediastinum. CT-guided biopsy revealed the tumor of vascular origin. The tumor was completely resected. Post-operative recovery was uneventful. After 14 months follow up patient is disease-free, and a CT scan of the chest showed no recurrence.

## Highlights

• A 54 years old female nonsmoker presented with a one-year history of epigastric pain.
• C-XRAY showed a large right hilar mass.
• CT Scan of thorax showed a large heterogeneous mass measuring 13.5 × 8.0cm.
• CT guided biopsy showed tumor of vascular origin.
• Radical surgical resection was performed with excellent outcome.

## 1 Background

Angiosarcoma is a rare malignant tumor originating from an endothelial cell. Most commonly, it occur as sporadic cutaneous lesions of the scalp, superficial connective tissues. Rarely do they involve deep soft tissues and body cavities. Weiss et al. reported after a review of 300 cases of Angiosarcoma that one-third of them occurs in the cutaneous layers, most commonly in the head and neck, one-fourth in the soft tissues, and rest in the liver, spleen, breast, bone, and heart [1,2]. Angiosarcoma is rarely encountered in the thoracic cavity. It can originate from Mediastinum, heart, and lung and is more commonly seen in the anterior Mediastinum. Mediastinal Angiosarcoma usually presents with chest pain or symptoms of its mass effect on surrounding structures, Angiosarcoma of the lung can be associated with diffuse alveolar hemorrhage [3,4]. A few cases of primary Angiosarcoma of Mediastinum have been reported in the medical literature. The Angiosarcoma is more commonly found in the anterior Mediastinum. We report a case of posterior mediastinal Angiosarcoma with rare presentation as epigastric pain. This case has been reported in line with SCARE criteria [5].

## 2 Case report

**Fig. 1:**
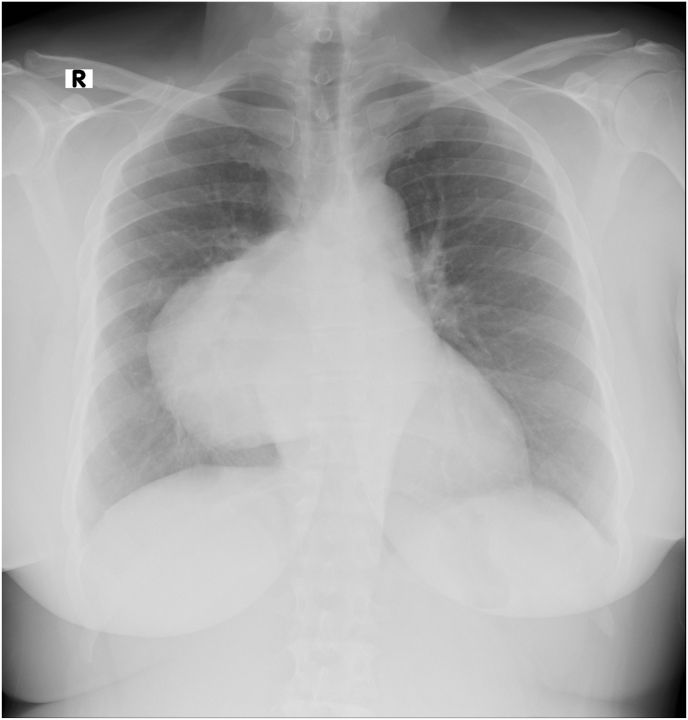
Chest X-Ray showing mass in right hemithorax.

**Fig. 2:**
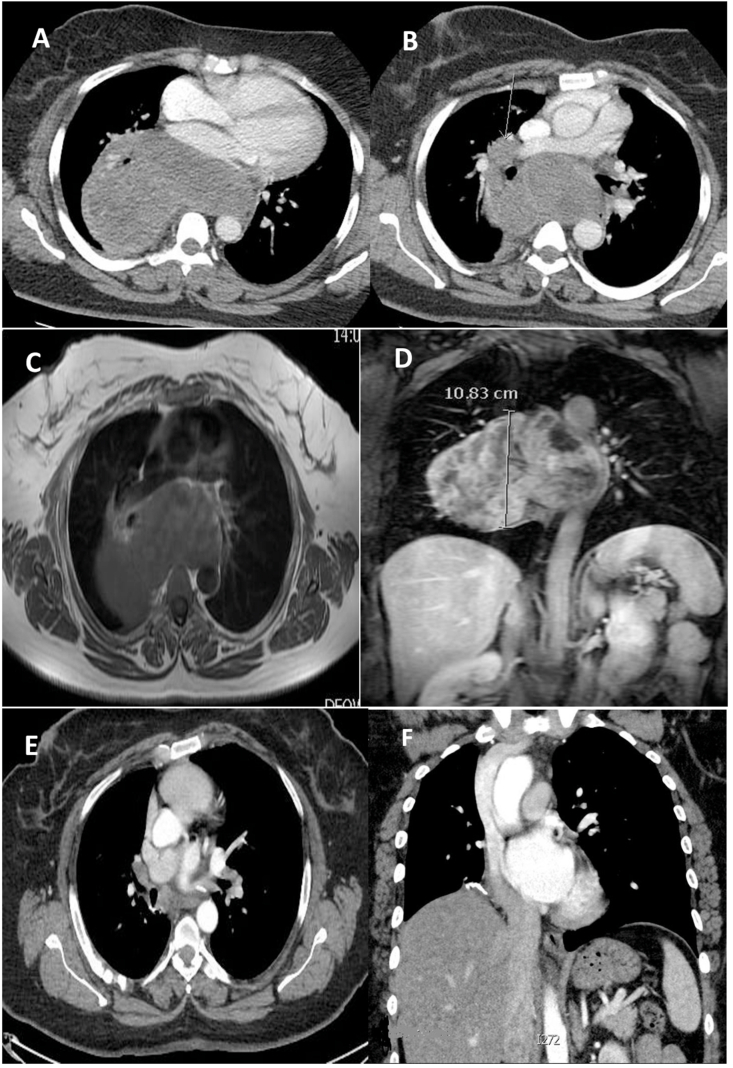
(A&B) A computed Tomographic scan of Thorax showed a large mass in the right hemithorax MRI (C&D) showing a large posterior mediastinal mass and it relation with vascular structures. (E&F) Post operative CT scan of Thorax 14 moths after surgery showing no recurrence.

**Fig. 3:**
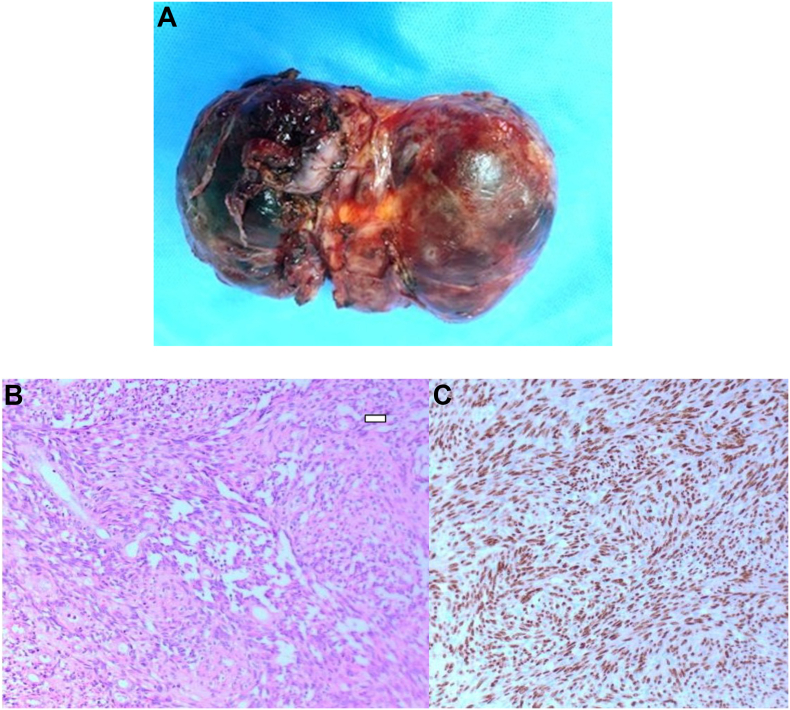
(A)Macroscopic image of resected specimen. (B) H&E4 x20 slide) Vasoformative neoplasm composed of anastomosing vascular channels, lined by mildly atypical endothelial cells. (C) ERG immunohistochemical stain slide) Diffuse nuclear staining of tumor cells by vascular marker ERG.

A 54 years old female nonsmoker presented with a one-year history of epigastric pain. There was no history of cough, shortness of breath, loss of weight, or appetite. On clinical examination, there was no jaundice, edema, or lymphadenopathy. She has no specific past medical history. On auscultation, there was decreased air entry in the right hemithorax. Her ultrasound of the abdomen and upper gastrointestinal endoscopy were reported normal by the referral hospital. Her basic blood investigations, white cell count, liver and renal panels were within normal range. Tumor markers including alpha-fetoprotein,beta-human gonadotropin (B_HCG)_, and Lactate dehydrogenase (LDH) were within normal limits. Her chest X-ray showed an increased right hilar shadow Fig. 1. A computed Tomographic scan of Thorax showed a large mass in the right hemithorax Fig. 1(A&B). MRI scan was done to further evaluate its relation to vascular structures Fig. 1(C&D). A multidisciplinary meeting advised to proceed for surgical resection. The tumor was approached through a right posterolateral thoracotomy, and there was a large tumor abutting to the pulmonary artery left atrium crossing the midline across the vertebral column. With meticulous dissection, the tumor was dissected carefully from the surrounding structures, pulmonary artery, aorta, oesophagus inferior pulmonary vein and left atrium and was completely excised. The chest cavity was irrigated with normal saline, a 32 F chest drain was placed in the pleural cavity, and thoracotomy closed in layers. The patient was extubated on a table and transferred to a high dependency unit in stable hemodynamic conditions for overnight monitoring. Her post-operative recovery was uneventful. On the second day, the chest drain was removed, and on the fourth day, she was discharged for further follow-up in outpatient. After a fourteen-month follow-up, a CT scan of Thorax showed no recurrence. Fig. 1 (D&E) Gross specimen showed a large tumor with two components Fig. 2 Histopathology report revealed that spindle cell neoplasm, low-grade Angio sarcoma. Fig. 3(A&B). Immunohistochemistry showed that CD31, CD34, and factor IV on the Ventana benchmark were positive, ERG stain was positive, and Pan-CK, S100, and desmin were negative.

## 3 Discussion

Angiosarcoma is a sporadic malignant tumor arising due to malignant proliferation and transformation of endothelial vascular cells. They are categorized into two groups spindle cell hemangioendothelioma and epithelioid hemangioendothelioma [6]. Primary mediastinal sarcoma comprises less than 10% of all mediastinal tumors. The incidence of Angiosarcoma is less than 1% of all soft tissue sarcoma. This tumor is commonly seen in the middle-aged population. Mediastinal Angiosarcoma usually arises from, right atrium, heart, lung, and pulmonary artery.[7] Mediastinal Angiosarcoma without any vascular origin has been reported.

Clinical presentation depends upon the Site of origin and its mass effect on the adjoining structures. Clinical manifestations are not specific, but such patients can present with cough, shortness of breath, hemoptysis, dysphagia, chest pain [8,9]. Rarely can they present as alveolar hemorrhage.

The exact etiology of this disease is not known. It may be related to iodine metabolism abnormalities, radiotherapy, use of glucocorticoids, environmental carcinogens exposure, arsenic, radiation, chronic lymphedema [10,11].

Although CT and MRI scans of the Thorax are used as diagnostic modalities for an initial assessment, the gold standard diagnostic tool is histopathology and immunohistochemistry. Even histopathology alone is sometimes is not sufficient to make a final diagnosis. Immunohistochemistry positive vascular markers factor VIII-related antigen CD31, CD34 can provide the firm diagnosis. [12] Wychulis et al. reported only 7 cases of Angiosarcoma after a retrospective study of 1046 patients of mediastinal tumors.[13] Pachter et al. l reported that in the thoracic cavity, most of the cases were found in the anterior Mediastinum. This neoplasm has a tendency of local invasion rather than distant metastasis.

Mediastinal Angiosarcoma has a poor prognosis due to aggressive biological behavior, and the five-year survival rate is 24%. Surgery is the best treatment if the tumor is resectable. The average survival is 3–6 months. Market al. reported a 5-year survival rate of 17% with surgery and adjuvant chemotherapy [14].

Currently, due to insufficient data and the rarity of these tumors, there is no established unified treatment protocol. Surgical resection and adjuvant radiotherapy, and chemotherapy is the common treatment modality. John et al.

Reported better long-term survival after radical surgical resection adjuvant radiation therapy [15]. Antiangiogenic gene-targeted therapy is a new modality with a promising outcome. However, the overall survival is very poor, and prognosis depends upon the size and degree of differentiation and presence of metastasis and resection margin [16,17]. Although radical surgical resection and adjuvant radiotherapy confer survival benefits, but due to the rarity and complexity of the biological behavior of this disease, further research is warranted to attain good survival.

## 4 Conclusion

Angiosarcoma of the posterior Mediastinum is a rare neoplasm with distinct pathologic features. We report a case of giant Angiosarcoma of posterior Mediastinum with unique presentation as epigastric pain. Radical surgical resection was performed, and resection margins were free of tumor. A Follow-up CT scan of the chest after 14 months showed no reoccurrence, and the patient is having a good quality of life.

## Ethical approval

IRB approval.

## Sources of funding

No source of funding

## Author contribution

Ikram ul Haq Chaudhry FRCS(CTh), operating surgeon and drafted the article.

Othman M Al Fraih, wrote abstract and case details.

Meenal A Al Abdulhai, structured abstract.

Ahmed Alshaer, highlights.

Burair Al Jassas, searched references.

Hisham Mamon images and legends.

Yousif AL Qahtani, wrote background.

Miral Mashour Histopathologist.

Abdullah M Al Ghamdi wrote part of discussion.

## Registration of research studies

1. Name of the registry: Research registry.
2. Unique Identifying number or registration 7368.
3. Hyperlink to your specific registration (must be publicly accessible and will be checked): http://www.researchregistry.com/browse-the-registry#home/.

## Consent

Written informed consent was obtained from the patient for publication of this case report and accompanying images. A copy of the written consent is available for review by the Editor-in-Chief of this journal on request”.

## Guarantor

Ikram ul haq Chaudhry.

## Provenance and peer review

Not commissioned, externally peer reviewed.

## Declaration of competing interest

No conflict of interest and there was no funding or financial assistance in this case.
